# Primary and secondary clustering of DSB repair foci and repair kinetics compared for γ-rays, protons of different energies and high-LET ^20^Ne ions

**DOI:** 10.1093/jrr/rrt210

**Published:** 2014-03

**Authors:** Martin Falk, Emilie Lukasova, Iva Falkova, Marie Davidkova, Alena Bacikova, Lenka Stefancikova, Lucie Jezkova, Jana Vachelova, Anna Michaelidesova, Alla Boreyko, Evgeny A. Krasavin, Stanislav Kozubek

**Affiliations:** 1 Institute of Biophysics, Academy of Sciences of CR, Brno, Czech Republic; 2 Nuclear Physics Institute, Academy of Sciences of CR, Řež, Czech Republic; 3 Institute of Chemical Technology Prague, Prague, Czech Republic; 4 Joint Institute for Nuclear Research, Dubna, Russia

**Keywords:** DNA double-strand breaks (DSB), DSB repair, chromosomal translocations, primary and secondary DSB clusters, higher order chromatin structure, ionizing radiation of different quality

## Abstract

**Purpose:** Ionizing radiations of different qualities (e.g. high-LET and low-LET) might differently interact with structurally and functionally distinct higher order chromatin domains (discussed in [ 1] and citations therein); this might be reflected by DNA double strand break (DSB) repair efficiency and the mechanism of how cancerogenous chromosomal translocations (CHT) form. Therefore, we compared the DSB repair kinetics and formation of γH2AX/p53BP1 repair clusters upon the action of γ-rays [ 2, 3], protons (15 and 30 MeV) [ 4], and ^20^Ne ions (preliminary data). Consequently, we discuss biological impacts of these clusters.

**Material and methods:** Immunostaining methods in combination with high-resolution confocal microscopy, performed on 3D-fixed normal human skin fibroblasts [ 2– 4], were used to study initial distributions of γH2AX and p53BP1 repair foci and their changes during the post-irradiation (PI) time, with a special concern on foci clustering. Irradiations with γ-rays, protons of different energies (15 and 30 MeV), and high-LET ^20^Ne ions was performed in IBP ASCR Brno (CR), NPI AVCR Řež (CR) and JINR Dubna (Russia), respectively.

**Results:** Upon irradiating cells with ^20^Ne ions, tracks of multiple clustered γH2AX and p53BP1 repair foci appeared immediately after the irradiation; these clusters, called here as the ‘primary clusters’, were rare in cells irradiated with γ-rays or protons (submitted). Though γH2AX/p53BP1 foci were positionally quite stable [ 2], ‘secondary clusters’ occasionally appeared after all kinds of irradiation during about 30 min PI. The formation of secondary clusters usually appeared due to the heterochromatin decondensation at the sites of heterochromatic DNA double-strand breaks (hcDSBs), followed by their protrusion into a limited space of nuclear subdomains of low density-chromatin (discussed in [ 1, 2, 5]).

**Conclusions:** Primary clusters appear in cell nuclei immediately PI as the consequence of highly localized energy deposition, while secondary clusters develop during (and because of) DSB repair. Primary DSB clusters probably represent the main cause of chromosomal translocations induced with high-LET radiations while secondary clusters seem to be more important for low-LET γ-rays and protons. Secondary clusters of primary clusters (higher-order clusters) observed for ^20^Ne ions might explain frequent formation of complex translocations upon the action of high-LET radiations. Finally, we suggest [ 1, 2, 4] a model that describes the relationship between the higher order chromatin structure, DSB formation, repair and misrepair.

## References

[1] Falk M, Lukasova E, Kozubek S (2010). Higher order chromatin structure in DSB induction, repair and misrepair. Mutat Res.

[2] Falk M, Lukasova E, Gabrielova B (2007). Chromatin dynamics during DSB repair. Biochim Biophys Acta.

[3] Falk M, Lukasova E, Gabrielova B (2008). Local changes of higher-order chromatin structure during DSB-repair. J Phys Conf Ser.

[4] Ježková L, Falk M, Falková I (2014). Function of chromatin structure and dynamics in DNA damage, repair and misrepair: γ-rays and protons in action. Appl Radiat Isot.

[5] Falk M, Lukášová E, Štefančíková L (2014). Heterochromatinization associated with cell differentiation as a model to study DNA double strand break induction and repair in the context of higher order chromatin structure. Appl Radiat Isot.

